# Gene Mutations and Genomic Rearrangements in the Mouse as a Result of Transposon Mobilization from Chromosomal Concatemers

**DOI:** 10.1371/journal.pgen.0020156

**Published:** 2006-09-29

**Authors:** Aron M Geurts, Lara S Collier, Jennifer L Geurts, Leann L Oseth, Matthew L Bell, David Mu, Robert Lucito, Susan A Godbout, Laura E Green, Scott W Lowe, Betsy A Hirsch, Leslie A Leinwand, David A Largaespada

**Affiliations:** 1 Department of Genetics, Cell Biology, and Development, University of Minnesota Twin Cities, Minneapolis, Minnesota, United States of America; 2 The Arnold and Mabel Beckman Center for Transposon Research, University of Minnesota Twin Cities, Minneapolis, Minnesota, United States of America; 3 Cancer Center, University of Minnesota Twin Cities, Minneapolis, Minnesota, United States of America; 4 Institute of Human Genetics, University of Minnesota Twin Cities, Minneapolis, Minnesota, United States of America; 5 Department of Integrative Physiology, University of Colorado, Boulder, Colorado, United States of America; 6 Genome Research Center, Cold Spring Harbor Laboratory, Cold Spring Harbor, New York, United States of America; 7 Cold Spring Harbor Laboratory, Cold Spring Harbor, New York, United States of America; 8 Laboratory Medicine and Pathology, University of Minnesota Twin Cities, Minneapolis, Minnesota, United States of America; 9 Department of Molecular, Cellular, and Developmental Biology, University of Colorado, Boulder, Colorado, United States of America; Stanford University School of Medicine, United States of America

## Abstract

Previous studies of the *Sleeping Beauty* (SB) transposon system, as an insertional mutagen in the germline of mice, have used reverse genetic approaches. These studies have led to its proposed use for regional saturation mutagenesis by taking a forward-genetic approach. Thus, we used the SB system to mutate a region of mouse Chromosome 11 in a forward-genetic screen for recessive lethal and viable phenotypes. This work represents the first reported use of an insertional mutagen in a phenotype-driven approach. The phenotype-driven approach was successful in both recovering visible and behavioral mutants, including dominant limb and recessive behavioral phenotypes, and allowing for the rapid identification of candidate gene disruptions. In addition, a high frequency of recessive lethal mutations arose as a result of genomic rearrangements near the site of transposition, resulting from transposon mobilization. The results suggest that the SB system could be used in a forward-genetic approach to recover interesting phenotypes, but that local chromosomal rearrangements should be anticipated in conjunction with single-copy, local transposon insertions in chromosomes. Additionally, these mice may serve as a model for chromosome rearrangements caused by transposable elements during the evolution of vertebrate genomes.

## Synopsis

Perhaps the greatest challenge for biomedical research in the post-genomics era will be to assign functions to the human set of ~25,000 genes. The classical method for discovering the gene function is mutation. Thus, technologies that can mutate genes in mammalian genetic models like the mouse are under development in hopes of creating an efficient method to complete this task. One such technology, the *Sleeping Beauty* (SB) transposon system, was developed for this purpose in 2001. This mobile DNA element is highly active in transgenic mice and has been shown to disrupt mouse genes efficiently. Geurts et al. describe a novel attempt to use the SB transposon in a forward-genetic screen using an insertional mutagen, the first attempt of its kind. They discovered that the process of transposon mobilization in mouse chromosomes can lead to dramatic effects on local genomic sequences. Indeed, transposons like SB can cause genomic rearrangements including deletions, inversions and translocations, involving tens of thousands to tens of millions of base pairs. This discovery has important implications for using transposable elements for mouse germline mutagenesis and, at the same time, may provide a model for studying genomic rearrangements that have helped shape vertebrate genomes during evolution.

## Introduction

The *Sleeping Beauty* (SB) transposable element is an effective tool for generating mutations in the germline [1–5] and somatic cells [6,7] of mice. Previous studies indicate that the advantages of this cut-and-paste transposon system are four fold. First, because the element is active in vivo, breeding transgenic mice is all that is required to generate heritable mutations. Second, insertional mutations in genes can be identified and tracked through generations using simple PCR-based techniques because the transposon vector serves as a molecular tag. Third, SB transposons have a strong tendency to reinsert during transposition at loci closely linked to their donor site. It has been proposed that this tendency will allow one to develop screens to saturate regions of the mouse genome that are syntenous to human genomic regions known to harbor disease genes [1,4]. A recent study achieved germline saturation mutagenesis by mobilizing chromosomally resident transposons in a region of mouse Chromosome 12 [5]. A fourth advantage is that the transposon vector can be designed to include functional elements that report expression patterns of mutated genes [4,8]. Previous strategies for the use of SB for germline mutagenesis have used reverse-genetic approaches, whereby potential mutations have been selected based on sequence information [1,8] or expression analysis [4,5]. The goal of the study presented here, rather, was to test whether the SB transposon system could be used as a mutagen for forward-genetic studies in the mouse germline.

Gene-dense regions, which share a high degree of linkage conservation with human chromosomal regions with disease genes, would be ideal for testing the SB system in this context. The Chromosome 11 region between the *Trp53* (69.3 Mb) and *Wnt3* (103.6 Mb) loci is very gene dense and is syntenous to a region of human Chromosome 17 known to harbor many disease genes [9]. The Inv(11)8Brd^Trp53–Wnt3^ strain of mice harbors an engineered inversion, generated using embryonic stem cell technology, between these two genes [10]. Kile et al. used the chemical agent *N*-ethyl-*N*-nitrosourea (ENU) to mutagenize this Chromosome 11 region of the mouse genome, taking advantage of this engineered chromosome as a “balancer” chromosome to facilitate identification of 88 recessive mutations, including lethals, between the *Trp53* and *Wnt3* loci [11]. Although this approach has uncovered interesting biology in this region of the genome [11,12], associating a single gene disruption with a phenotype has been a challenge due to the lack of a molecular landmark for identifying the ENU-induced mutation. The 88 reported mutations, however, provide an opportunity to evaluate the results of a saturation mutagenesis screen using transposons in the same region of the genome and potentially to assign specific genes to similar phenotypes, because the transposon serves as a molecular tag for mutation.

Using a donor site of mutagenic transposon vectors on mouse Chromosome 11, near the 90-Mb position, we tested the SB transposon system in a three-generation, forward-genetic, regional mutagenesis screen for recessive-lethal and viable phenotypes. Here we report the recovery of visible and behavioral mutants, a high rate of recessive lethal phenotypes, and the identification of alternative mechanisms of transposition-mediated mutation. We also compare our experiences to the ENU-based mutagenesis of the same region [11,12] and the aforementioned SB transposon-induced regional mutagenesis of mouse Chromosome 12 [5].

## Results

### Generation of Mice for a Forward-Genetic Screen

The protocol for SB-mediated germline mutagenesis has been described by multiple groups [2,3,13]. Briefly, two mouse strains, one transgenic for a mutagenic SB transposon vector (mutator), and the other strain transgenic for a SB transposase expression construct (jumpstarter), are bred together to generate doubly transgenic “seed” mice. The transposase mobilizes transposons in developing gametes of seed mice, and the events are recovered in an outcross generation.

**Figure 1:**
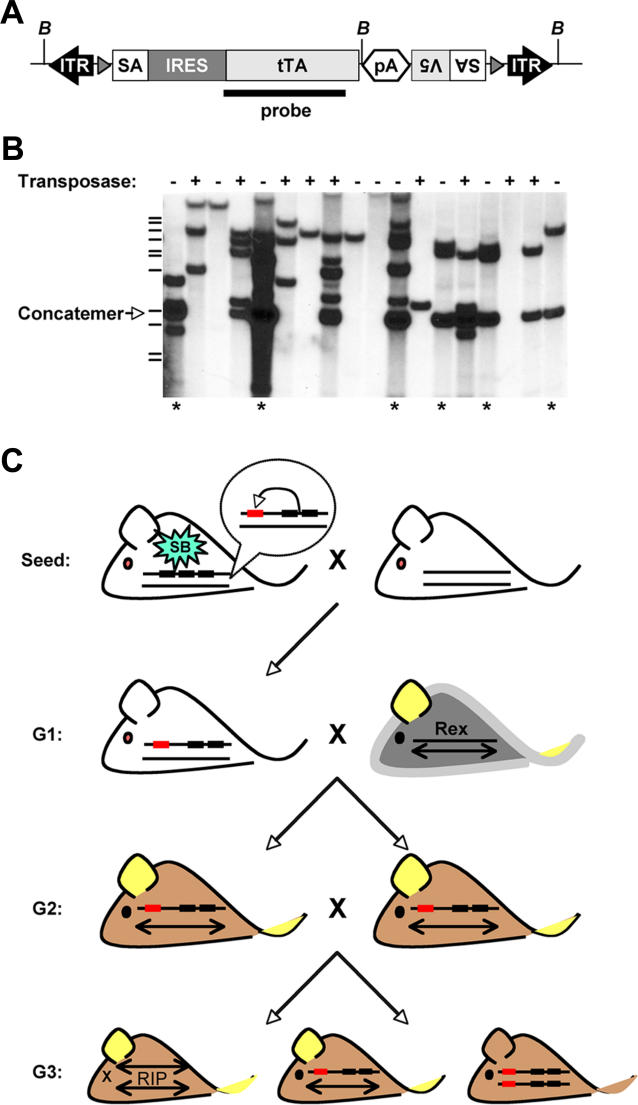
Design of a Forward-Genetic Screen (A) The T2/GT3/tTA gene-trap tTA transposon was designed with splice acceptors (SA) in both orientations and the bidirectional SV40 polyadenylation signal (pA) to truncate expression of an endogenous gene after insertion into an intron. LoxP recombination sites (gray arrowheads) flank the mutagenic core of the transposon to potentially rescue a transposon-induced mutation. (B) Southern blot and PCR analysis (+/–, top) was used to identify G1 animals that inherited the concatemer, but not the transposase transgene (asterisk). (C) Insertions (red rectangle) genetically linked to the concatemer donor site (black rectangles) on Chromosome 11 (—) are homozygosed in a three-generation breeding scheme using the Inv(11)8Brd^Trp53–Wnt3^ strain balancer chromosome (↔) with its engineered *Wnt3* mutation and visible *Agouti* marker conferring a yellowish color to the ears and tail. G1 animals were crossed to mice that carry a balanced *Rex* (curly coat) mutation (gray outline). Animals inheriting two copies of the balancer die in utero.

The T2/GT3/tTA “gene-trap tTA” vector (Figure 1A) and generation of transgenic mice is described elsewhere [8]. Founder line 6660 was previously mapped to chromosome 11, band 11C, by fluorescence in situ hybridization (FISH), and harbors a concatemer of approximately 30 copies of the transposon vector [8]. We renamed this line the “GT3A” line and used two different strains of transgenic mice to mobilize these transposons from their Chromosome-11 donor site in the germline of mice. Using the CAGGS-SB10 strain [2] as a source of transposase, we can achieve germline mobilization rates of nearly three events per gamete [8], whereas we report here that the RosaSB11 strain [7] can mobilize the T2/GT3/tTA transposon at a rate of one insert per gamete (unpublished data). We also verified by progeny testing and FISH that phenotypically normal homozygous GT3A offspring could result from an intercross of hemizygous carriers (unpublished data).

### Design of a Screen to Mutagenize Mouse Chromosome 11

Transposase-negative offspring of seed mice that inherited the transposon donor site by Southern blot (Figure 1B) were designated with a pedigree letter and entered as first-generation animals into a three-generation breeding scheme (Figure 1C). We took advantage of the Inv(11)8Brd^Trp53–Wnt3^ strain of mice to preferentially homozygose local transposon-induced mutations in Chromosome 11 between *Trp53* and *Wnt3*. Carriers of the balancer chromosome are identified by expression of an *Agouti* transgene incorporated into one breakpoint of the engineered inversion [11]. Additionally, animals inheriting two copies of the balancer chromosome die in utero because one inversion breakpoint is in the essential *Wnt3* gene [11].

The results of the ENU screen [11,12] gave insight into the phenotypes we might expect to recover, and importantly, might be rare to discover in a screen. Recessive lethal (63%), neurological (11%), skeletal (6%), and growth/size defects (6%) made up the majority of recovered phenotypes in that study [11]. Anticipating recovery of a similar range of phenotypes, a comparable phenotype screen was designed to recover mutations resulting in growth/size, neuromuscular, or behavioral phenotypes and to identify craniofacial or limb defects (see Materials and Methods). Two control pedigrees, generated in the absence of the transposase strain, were randomly introduced into the screen and were found to be phenotypically normal. Thirty-eight pedigrees were screened using this strategy, resulting in one visible, one behavioral, and 21 recessive, prenatal lethal phenotypes (Table 1).

**Table 1:**
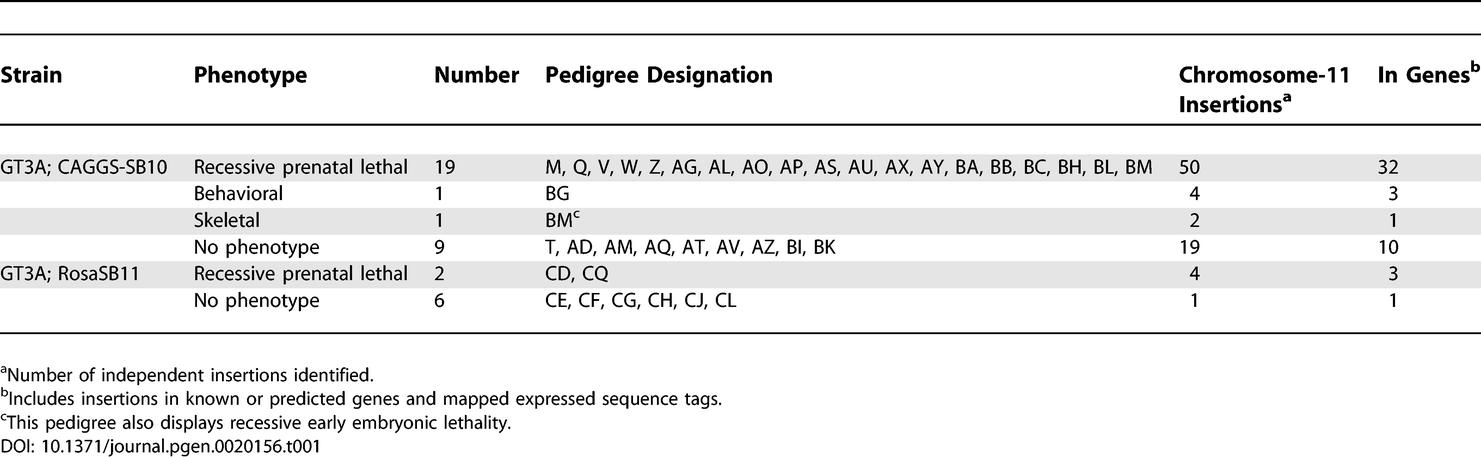
Pedigree Phenotype and Cummulative Insertion Data

### Local Transposition and Gene Mutation on Mouse Chromosome 11

**Figure 2:**
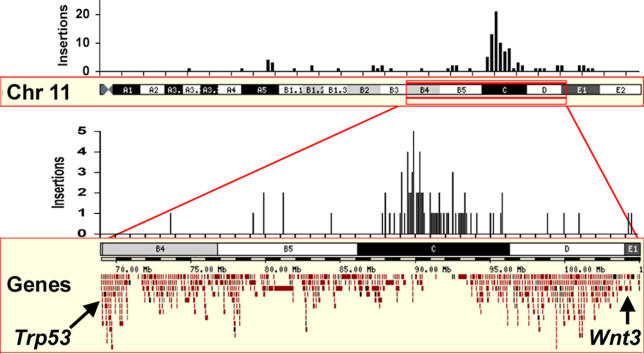
Distribution of Chromosome 11 Insertions The insertions over the entire Chromosome 11 and the gene-dense, balanced region between *Trp53* and *Wnt3* are shown as a histogram over the ENSEMBL *ContigView* (http://feb2006.archive.ensembl.org/Mus_musculus/contigview?region=11&vc_start=69.3M&vc_end=103.6M&h=11). The number of insertions over the whole chromosome is shown in 1-Mbp bins, while the balanced region is shown in 100-kb bins.

Transposon insertions in the genomes of mutant mice were characterized using previously described methods [6] to measure progress on saturating this gene-dense region with mutations and to catalog any gene disruptions. The NCBI m34 build (May 2005 freeze) was used to identify the genomic position of 175 unique T2/GT3/tTA insertion sites using the BLAST function of the ENSEMBL genome browser http://www.ensembl.org/Mus_musculus/blastview. A full description of the genomic position for the local and genome-wide insertions can be found in Table S1. As predicted, 104 insertions (59%) were localized to mouse Chromosome 11 and, of those, 75 mapped to the balanced region between *Trp53* and *Wnt3* (Figure 2). In total, 67% (50/75) of insertions within the balanced region were inserted into 38 different known or predicted genes (Table S1).

### Dominant Polysyndactyly and Recessive Hyperactivity Phenotypes of Mutant Pedigrees

**Figure 3:**
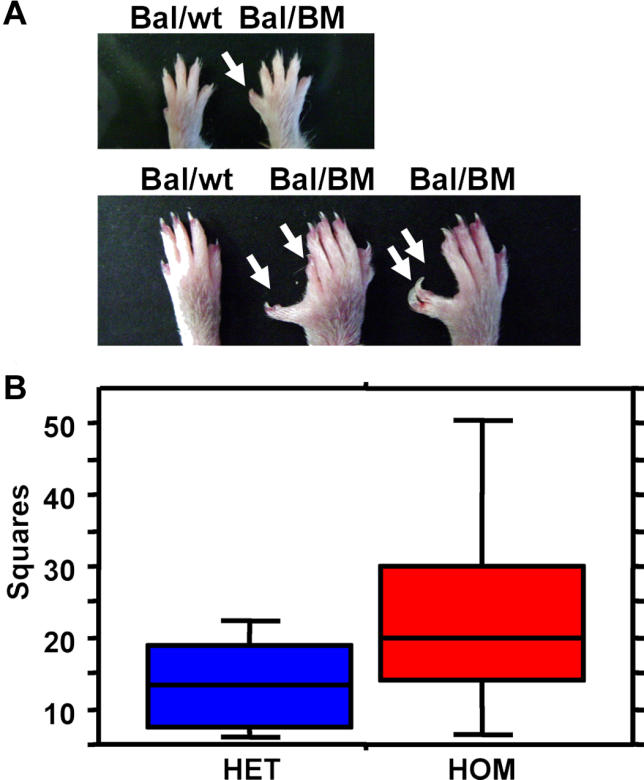
Visible and Behavioral Phenotypes as a Result of Transposon Mutation (A) Dominant polydactyly (extra digits) or polysyndactyly (extra, fused digits) is evident in the fore (top) and hind limbs (bottom) of animals in pedigree BM. (B) A recessive hyperactive phenotype is measured by the number of squares visited in the SHIRPA arena (see Materials and Methods) in homozygous animals in the viable pedigree BG (*p* = 0.0113 by unpaired t-test).

Dominant polysyndactyly is evident in carriers of the mutagenized Chromosome 11 in pedigree BM (Figure 3A). These mice demonstrate duplication and often fusion of the anterior digit on the fore and hind limbs. The open field test, performed in the SHIRPA arena (see Materials and Methods), identified a recessive hyperactivity phenotype as homozygous BG animals traveled through more squares when compared to their control siblings (Figure 3B).

Candidate gene insertions were identified in the mutagenized Chromosome 11 of pedigrees BM and BG. Heterozygous pedigree BM mice harbor an insertion (03A-0204, Table S1) in a pioneer gene (UniProt/TrEMBL ID Q5SRA9). This gene is highly conserved throughout all major animal phyla, yet no clues as to its function have been reported in other model organisms. Homozygous pedigree BG animals display the hyperactive phenotype and harbor an insertion (03A-0355, Table S1) in a member of a family of genes known to be expressed in the nervous system and that have been associated with neurological disorders. We are currently attempting to correlate these candidate single-gene disruptions on Chromosome 11 with phenotypes. However, subsequent analysis of the recessive prenatal lethal phenotypes led us to discover that other types of mutations were present on Chromosome 11 in these and other pedigrees, as is described below. Thus, it is possible that transposon insertions are not responsible for these viable phenotypes.

### Recessive Lethality as a Result of Transposon Mutagenesis of Chromosome 11

As mentioned above, 21 of the 38 pedigrees demonstrated recessive lethality, all of which were prenatal lethal (Table 1). While we anticipated a high rate of recessive lethal phenotypes, this result was in stark contrast to the ENU study where out of 55 (recently updated to 59 [12]) recessive lethal phenotypes isolated, only 30 were characterized as prenatal [11]. Based on the initially reported 55 of 88 balanced ENU-induced mutations resulting in lethal phenotypes [11], this region of the genome may harbor a higher than average density of essential mouse genes. SB transposition-induced lethal pedigrees harbored nearly twice as many potential gene disruptions per pedigree on average than non-lethal pedigrees (1.7 vs. 0.9) (Table 1). One possibility is that the germline mobilization rate is sufficiently high such that the screen would identify lethal mutations at this rate. We addressed this hypothesis by both analyzing candidate gene disruptions and complementation testing between lethal pedigrees.

**Figure 4:**
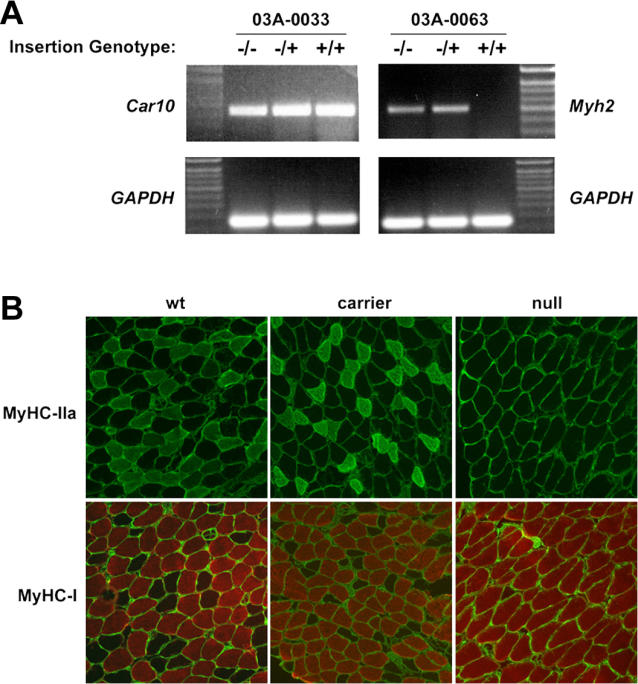
Molecular Analysis of T2/GT3/tTA Gene Disruptions (A) RT-PCR analysis of wild-type (−/−), hemizygous (−/+), and homozygous (+/+) carriers of insertions 03A-0033 and 03A-0063 are compared. Primers to the housekeeping *Gapdh* gene were used as an internal control for sample quality. (B) Immunohistochemical staining of wild-type (wt), carrier, and null soleus muscles for MyHC type IIa (top) and type I (bottom). Samples were co-stained with Anti-Laminin to outline individual fibers.

Candidate gene insertions in lethal pedigrees were examined for their association with lethal phenotypes. Carriers were repeatedly outcrossed until recombinants were obtained separating pedigree M insertion 03A-0033, in the mouse carbonic anhydrase 10 gene *(Car10),* and pedigree V insertion 03A-0063, in the mouse myosin heavy chain 2 gene *(Myh2)* (Table S1), from all other insertions and the donor site (unpublished data). Carriers of the isolated insertions were intercrossed to discover any associated recessive phenotypes. Homozygous carriers of either insertion were fertile, healthy mice, and displayed no visible or behavioral abnormalities in our assays, showing that the lethality in these pedigrees was not due to transposon insertions in these genes. These gene trap insertions were subsequently characterized to determine their effect on gene expression. Figure 4A shows the RT-PCR analysis on tissues from wild type, carrier, and homozygous animals for each insertion. Insertion 03A-0033 does not appear to alter expression of *Car10,* although insertion 03A-0063 ablates expression of *Myh2* at the level of transcription. We have previously published that the carp ß-actin splice acceptor in the T2/GT3/tTA does not always function to intercept splicing as designed [8] and is apparently defective in disrupting *Car10* expression here. The reverse orientation splice acceptor (Figure 1A), derived from the mouse hypoxanthine phosphoribosyltransferase gene [8], however, is effective in the case of the *Myh2* insertion.

*Myh2* encodes the myosin heavy chain IIa peptide (MyHC-IIa) and is one of three adult fast skeletal muscle myosin genes clustered on mouse Chromosome 11 [14]. Immunohistochemical staining of the soleus muscle from homozygous carriers of insertion 03A-0063 demonstrate lack of MyHC-IIa in muscle fibers, compared to carrier and wild-type sibling controls (Figure 4B). Null mutations in the other two Chromosome-11 fast muscle myosins, IIb [15], and IId [16], are also viable, but they demonstrate severe muscle pathology. It was demonstrated in those studies that neighboring Chromosome-11 MyHC genes can compensate for the loss of expression of one MyHC in mutant mice, though they are not functionally redundant [14]. Figure 4B presents evidence that MyHC genes on other mouse chromosomes can compensate as well since type I MyHC, encoded by the *Myh7* gene on mouse Chromosome 14, is upregulated in IIa mutant muscle.

### Complementation Testcrosses Reveal Major Complementation Groups

**Figure 5:**
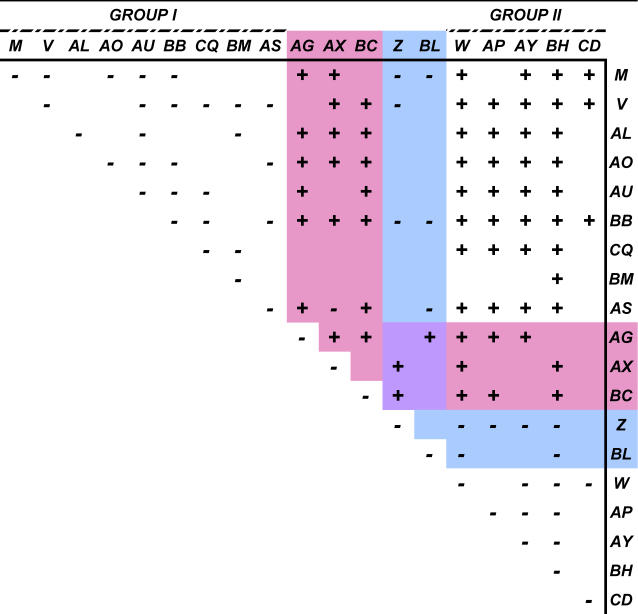
Complementation Testcrosses For each testcross, heterozygous animals from independent lethal pedigrees (see Table 1) were intercrossed to obtain offspring that inherited both copies of their respective mutagenized chromosomes as detected by PCR genotyping or balancer screening (see Figure 1B). Noncomplementation (−) and complementation (+) divided the lethal pedigrees into at least six complementation groups, with two major groups labeled I and II. Pedigrees highlighted in pink complemented every other pedigree tested except one case, where AX failed to complement AS. Pedigrees in blue failed to complement pedigrees other than AG, AX, and BC (purple).

To assess whether any of the lethal pedigrees were allelic, complementation testcrosses using G2 animals were performed. ENU mutagenesis in the same region of the genome revealed 23 complementation groups among 24 lethal pedigree testcrosses [11]. In contrast, most of the pedigrees generated by transposon mutagenesis fell into one of two major complementation groups (designated I and II, Figure 5), suggesting that most of our lethal pedigrees were the result of one of two mutations occurring repeatedly in our screen. Interestingly, pedigrees Z and BL failed to complement all pedigrees except AG, AX, and BC, which complemented all pedigrees tested with the single exception being that AX did not complement pedigree AS. Pedigrees Z and BL, therefore, have mutations that overlap with both major complementation groups, while AS likely has more than one lethal mutation since it fails to complement group I pedigrees and, separately, pedigree AX. Pedigrees AG and BC have unique lethality-inducing mutations. Additionally, pedigrees CQ and CD, generated with the RosaSB11 source of transposase, fell into groups I and II, respectively, suggesting that neither reducing the transposition rate, nor using the catalytically enhanced SB11 transposase [17], can eliminate the incidence of the mutations arising in the two major complementation groups. Having cloned many insertions from these mice, the prevalence of two major complementation groups could not be explained by the repeated disruption of the same essential mouse genes (Table S1). Thus, we investigated whether other types of mutations might occur near the donor site to explain these observations.

### Microdeletions, Inversions, and Chromosomal Translocations as a Result of Transposition

**Figure 6:**
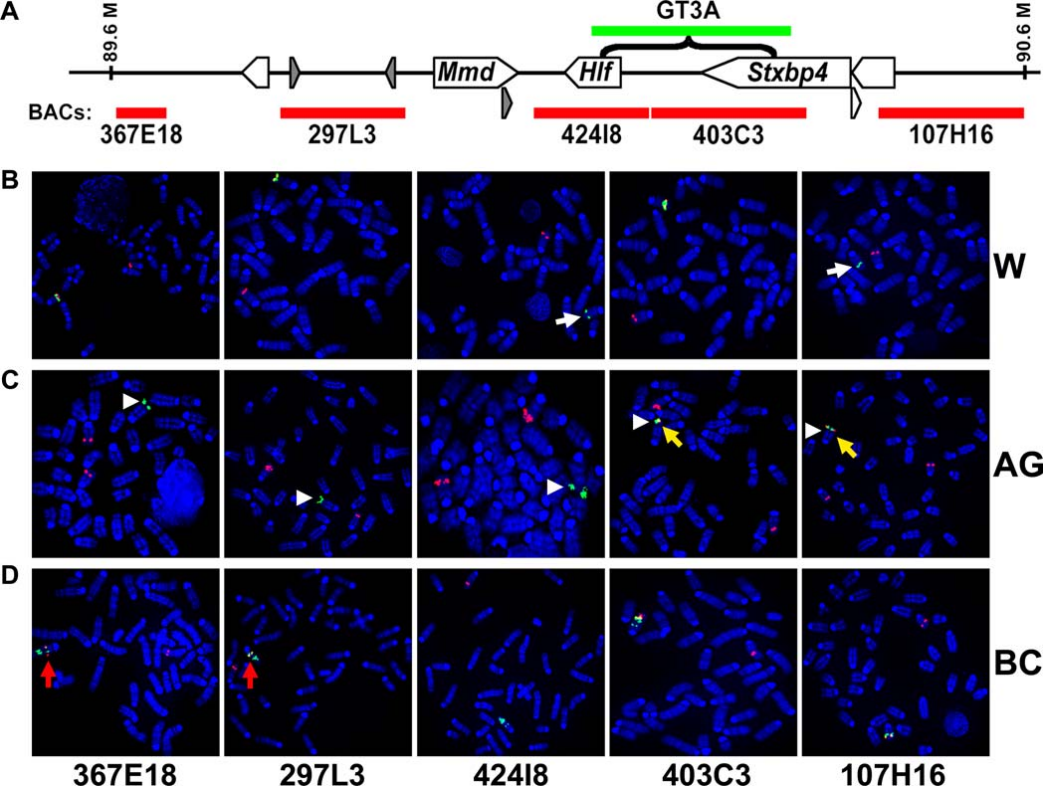
Molecular Evidence of Chromosomal Rearrangements after Transposition (A) Five BAC probes (red bars) were designed to the Chromosome 11 region from approximately 89.6–90.6 Mb (ENSEMBL m34 build, May 17, 2005 freeze). The transposon donor site (green) is presumed to be within the bracketed area based on the accumulation of insertions in this region. Single copies of the transposon were not detectable in this assay. Representative FISH hybridizations to metaphase preparations are shown. (B) Evidence for deletion of BAC signals 424I8 and 107H16 in pedigree W (white arrows). (C) Translocation of transposons (white arrowheads) along with distal Chromosome 11 sequence (yellow arrows) in pedigree AG. (D) Evidence for inversion of a large region of Chromosome 11 is detected by BAC probes 367E18 and 297L3 (red arrows) in pedigree BC, likely involving multiple copies of the transposon.

To determine whether local genomic rearrangements are present near the donor site of mutant chromosomes, FISH was used to probe control and mutant pedigree chromosomes. Several bacterial artificial chromosome (BAC) clones were obtained spanning a 1-Mb region from 89.6–90.6 Mb (according to ENSEMBL m34 build, May 17, 2005 freeze) and flanking the presumptive donor site based on insertion data (Figure 2). Each of five BAC clones (Figure 6A), detected with rhodamine (red), was co-hybridized with a fluorescein-detected (green) transposon probe to metaphase chromosome preparations. Red signals for each probe were present near the green donor site of GT3A control mice, suggesting that none of the regions covered by the probes were deleted in the initial transgenesis (unpublished data).

Hybridization of pedigree W demonstrated a loss of signal from BAC probes 424I8 and 107H16 (Figure 6B, white arrows) when compared to the normal copy of Chromosome 11, suggesting a loss of more than 300 kb of genetic material adjacent to the donor site. Pedigree AG was previously confirmed to be a lethal pedigree because homozygous carriers of an insertion near the concatemer donor site (03A-0071, Table S1) cannot be generated (*p* <0.01 by χ^2^ analysis, unpublished data). BAC probing revealed a sizeable insertion involving a portion of the concatemer (Figure 6C, white arrowheads), and at least 400 kb of distal Chromosome 11 sequence overlapping probes 403C3 and 107H16 (yellow arrows), into distal Chromosome 5 as determined by spectral karyotyping (unpublished data). Thus, these mice have two normal copies of Chromosome 11, which explains why pedigree AG complements all other pedigrees (Figure 5). This is the second example of insertion of the GT3A concatemer into another chromosome, as we previously reported the insertion of a large portion of the concatemer into Chromosome 4 and subsequent local transposition from the translocated donor site to a nearby gene [8]. A third mutant pedigree revealed a large inversion covering tens of millions of base pairs. Multiple green transposon signals can be observed in pedigree BC, suggesting multiple copies of the gene-trap tTA vector were involved in complex rearrangements since a single copy of the transposon could not be detected by this probe (unpublished data). One rearrangement situated the red signals for probes 297L3 and 367E18 to a more proximal position along Chromosome 11 when compared to the normal copy (Figure 6D, first two panels). Based on the physical distance of the probes, this inversion likely encompasses more than 50 million base pairs.

**Figure 7:**
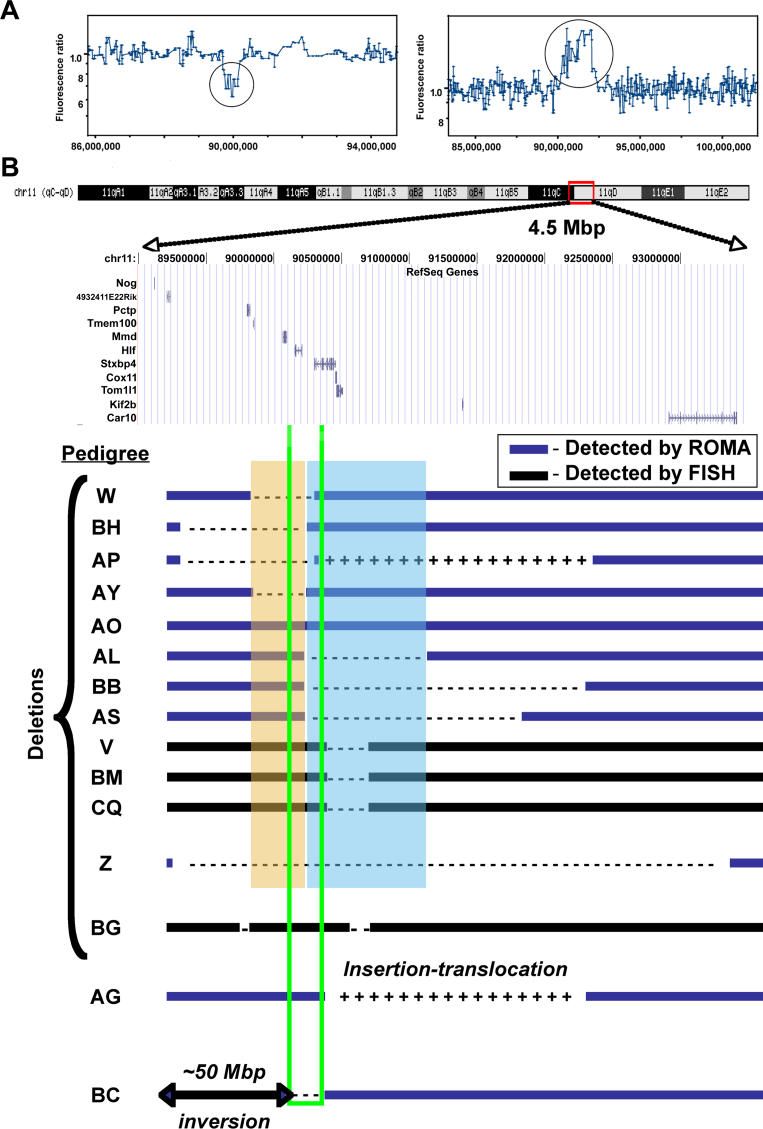
Defined Genomic Rearrangements Caused by Transposition (A) Moving average plots of ROMA data of deletion in pedigree W and amplification (due to insertion) in pedigree AG. (B) Summary of rearrangements as determined by FISH (black bars) or ROMA (blue bars). The green box represents the concatemer, though its position relative to the deletions is speculative. ROMA detects loss (---) of chromosomal material, and defines the minimal overlapping regions for complementation groups 1 (blue box) and 2 (orange box), as well as amplification (+++) of genomic sequences. Where the FISH method was used (black bars) the true extent of each rearrangement could not be determined.

The representational oligonucleotide micro array (ROMA) technique was developed to identify genome-wide copy number variation in tumor DNA [18]. ROMA analysis of mutant pedigree genomic DNA verified that, although the rest of the genome was unaffected (unpublished data), a reduction in copy number of a portion of mouse Chromosome 11 was seen in pedigree W and an amplification of Chromosome-11 sequences was seen in pedigree AG (Figure 7A). Based on the UCSC mm5 build (May 2004 freeze), pedigree W deletion extends from approximately 89.7–90.1 Mb, a loss of ~400 kb of sequence, while the amplification (due to insertion of sequences from Chromosome 11, Figure 6C) in pedigree AG was a roughly 1.9-Mbp region from ~90.3–92.2 Mb. This insertion is detected as amplification by ROMA because the region of Chromosome 11 that would have shown a deletion was bred away. Thus, pedigree AG animals now have two normal copies of Chromosome 11 in addition to the inserted 1.9-Mbp region into Chromosome 5.

Figure 7B summarizes ROMA and FISH data collected for Chromosome 11 rearrangements from various pedigrees. We propose that the two major complementation groups (Figure 5) can be explained by overlapping deletions that occurred during transposition. Complementation group I, represented by pedigrees AL, BB, AS, V, BM, and CQ, carry overlapping deletions detectable by ROMA (blue bars) or FISH (black bars) in a region of Chromosome 11 immediately distal to the predicted concatemer donor site (Figure 7B). This region harbors the syntaxin binding protein 4 gene, and mouse homologs of a yeast cytochrome C oxidase assembly protein, and of the chicken target of myb1-like 1 *(Tom1l1)* genes (blue box, Figure 7B). Even though it is a member of complementation group I, pedigree AO did not harbor a deletion detectable by the ROMA technique. This pedigree, rather, harbors multiple transposon insertions, one within the non-complementation region just defined by the pedigrees above, situated in *Tom1l1* (insertion 03A-0297, Table S1), and may indicate that *Tom1l1* is essential.

In contrast to those distal mutations caused by transposition, pedigrees W, BH, AP, and AY, members of complementation group II, have overlapping deletions which are proximal to the predicted donor site. This region contains the monocyte to macrophage differentiation-associated and hepatic leukemia factor genes (orange box, Figure 7B). ROMA analysis of pedigree AP also identified a tandem duplication of Chromosome-11 sequences distal to the donor site (Figure 7B).

The ~4.3-Mbp deletion in pedigree Z, detected by ROMA, explains why this pedigree fails to complement either group I or group II pedigrees, because this larger deletion would cover both of the above regions. Additionally, the non-lethal pedigree BG contains two separate deletions not immediately adjacent to the donor site, minimally defined by FISH, suggesting that these regions do not harbor essential sequences. Two cases of insertion translocations have been identified as demonstrated in pedigree AG (Figure 6C) and a previously described translocation involving just the transposon concatemer [8]. Finally, pedigree BC harbors both inversions and deletions, defined by FISH (Figure 6D) and ROMA (Figure 7B). In summary, out of nine examined cases of transposon mobilization on Chromosome 11, every pedigree demonstrated some form of genomic rearrangement, spanning hundreds of thousands to tens of millions of base pairs.

### Genome Rearrangements in Somatic Cells of a GT3A; RosaSB11 Mouse

**Figure 8:**
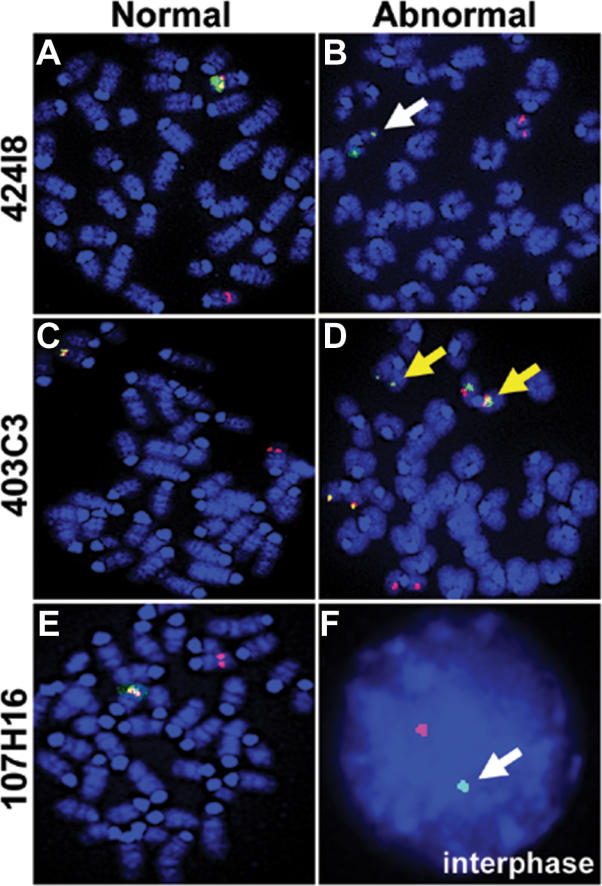
De Novo Rearrangements in Somatic Cells of a GT3A; RosaSB11 Mouse Metaphase and interphase FISH images of normal (A, C, E) and abnormal (B, D, F) splenic lymphocytes from a doubly transgenic mouse are shown using the same probes as Figure 6. Evidence for deletion (white arrows) and translocated Chromosome 11 sequences (yellow arrows) were evident for these three probes. These data, including other probes, are further summarized in Table S2.

To determine whether genomic rearrangements similar to those inherited in our germline screen could be detected in somatic cells, we probed cell preparations from a GT3A; RosaSB11 mouse and a control GT3A mouse by FISH using the same BAC probes as above. Screening interphase and metaphase chromosomes revealed a 40% frequency of signal loss from BAC probe 424I8 (Figure 8), suggesting this region is frequently deleted or a clone of deleted cells existed in the spleen. A background of approximately 7–10% probe non-detection existed when probing control GT3A cells with any BAC or transposon probe due to technical artifacts associated with the FISH method. Loss of signal 107H16 could be verified at a low frequency because the red 107H16 probe signal was not adjacent to the green transposon signal in some interphase cells (Figure 8). One rare cell also demonstrated translocation and possible amplification of signal from probe 403C3 (Figure 8). These data, summarized in Table S2, suggest that chromosomal rearrangements and deletions can be readily identified in somatic tissues when transposons and transposase are present in the same cell.

## Discussion

We have investigated the utility of the SB transposon system as a forward-genetic tool in the mouse germline to recover a range of lethal and viable phenotypes. In a limited number of pedigrees, we recovered one dominant limb and one recessive behavioral phenotype. Unexpectedly, however, genomic rearrangements including deletions and inversions near the transposon donor site, as well as insertion of the donor site plus nearby Chromosome-11 sequences into other chromosomes, caused a high frequency of early embryonic phenotypes in our screen. We hypothesize that the donor site of transposons is situated between these two sets of deletions (Figure 7B, blue and orange boxes), and that transposition resulted in deletions that were proximal or distal to the donor site. We further hypothesize that sequences contained within these deleted regions are essential, and thus result in the recessive lethal phenotypes we observed. We also observed similar de novo rearrangements in somatic cells. These genetic changes may not have been previously recognized because earlier studies involved sequence- or expression-driven approaches. Through a forward-genetic approach, we identified a high frequency of recessive lethal mutations, and then by complementation testing and molecular analysis, determined the nature of the lesions.

Small- and large-scale local genome rearrangements including insertions, deficiencies, duplications, inversions, and translocations are a common result of *Ac/Ds* [19–21], *Tam3* [22], Tc1 [23], *P* [24,25], and bacterial [26] element transpositions. Although we have not speculated on the mechanisms by which SB transposons cause rearrangements, the mechanisms for those elements, representing three major families of eukaryotic cut-and-paste transposons, are well-characterized in those reports. Genomic rearrangements caused by alternative transposition of *P* [25] and *Ac/Ds* [19–21] elements are particularly well understood and the involvement of the latter in altering the structure of *Maize* genes is now known [27]. The SB-induced genomic rearrangements reported here could be caused by alternative mechanisms of transposition, or due to chromosome instability caused by double-strand breakage during excision and integration. In either case, these events may have been exacerbated by the fact that the GT3A donor site consists of many identical copies of the transposon elements and the high mobilization rate in the GT3A strain.

This discovery has implications for using SB and possibly other transposable elements as mutagenic tools in the mouse germline and soma. A recent study took advantage of the local hopping phenomenon of SB transposons to demonstrate regional saturation mutagenesis [5]. Employing a polyA-trap design, insertions were cloned from sperm and identified in every gene over a 4-Mb interval. The data presented here suggest other genetically linked lesions often accompany insertions in local genes. Indeed, single gene insertions were identified in the cases of the dominant polysyndactyly and recessive hyperactivity, seen in pedigrees BM and BG, respectively. Further analysis, however, led us to discover genomic deletions within the mutagenized Chromosome 11 in each of these pedigrees that are also genetically linked to these phenotypes. Pedigree BM, along with the polysyndactyly phenotype, is a member of complementation group 1 (Table 1, Figure 7B) and harbors a deletion distal to the concatemer donor site. The hyperactive pedigree BG contains two separate deletions (Figure 7B) in addition to single copy insertions. Since these genomic rearrangements and transposon insertions are genetically linked to the phenotypes we observed, we cannot rule out that the deletions are not causative. As is always the case, it will be crucial to prove that any transposon insertion causes the observed phenotype by remobilizing it from within the gene by re-exposure to transposase, by removing the mutagenic elements of the vector, or transgene rescue. We have previously reported that the germline mobilization rate of single-copy elements is only about 1%, and thus loxP recombination sites were engineered into the T2/GT3/tTA transposon (Figure 1A), flanking the mutagenic core of the transposon, to potentially rescue expression of a gene [8].

Somatic transposition of oncogenic SB transposons is a powerful method for studying the cancer genome of tumors previously inaccessible to retroviral insertional mutagens [6,7]. We have aged several GT3A; SB11 mice and have seen no statistically significant increase in mortality compared to controls. It will be important, however, to closely examine whether continuous transposon mobilization leads to genomic rearrangements that can contribute to cancer development in these models. Additionally, although *Sleeping Beauty* was the first vertebrate transposon system shown to be active in the germline and somatic cells of mice in vivo, the fly transposon *Minos* [28] and the lepidopteran *piggyBac* element [29] were more recently developed for these purposes. *Minos,* like SB, belongs to the Tc1/*mariner* superfamily of transposons and, although *piggyBac* is in a family of its own, due to their cut-and-paste mechanism of mobilization and local hopping activities, they likely have the potential to cause similar genomic rearrangements as we have reported on here. While analyzing the results of mobilizing these vectors, it will be important to include methods to detect these mutations.

Finally, we propose that the genomic rearrangements caused by mobilization of SB transposons may serve as a model for the genomic rearrangements that have occurred during the evolution of vertebrate genomes. Major rearrangements that lead to the observed conserved blocks of synteny between mouse and human chromosomes are consistent with random chromosomal breakage [30]. Mainstream hypotheses, however, correlate smaller scale rearrangements, including insertion, deletion, inversion, and duplications within chromosomes, with the amplification and activities of transposable elements [31–33] though most attention has been given to copy-and-paste elements. To our knowledge, this is the first report of local genomic rearrangements caused by a cut-and-paste transposon in a vertebrate. As far as is known, cut-and-paste transposons have been inactive in mammalian genomes for tens of millions of years [34,35]. Nevertheless, cut-and-paste elements do lead to genomic rearrangement in nature, as endemic inversions in isolated populations of *Drosophila* have been attributed to *hobo*-element cut-and-paste activity [36]. We have shown here that the high frequency of transposition of the teleost fish-derived SB element [37] in transgenic mice can cause similar rearrangements at high frequency. Though the synthetic SB element is likely to be much more active than an endogenous cut-and-paste element would have been millions of years ago, we postulate that analogous rearrangements could contribute to the speciation and evolution of vertebrates. Such mutations by a highly active element would undoubtedly be deleterious to a host species over several generations. It now seems plausible that genomes evolved mechanisms to suppress cut-and-paste elements to protect against these damaging rearrangements rather than to prevent the accumulation of rare mutations caused by single-copy insertions.

## Materials and Methods

### Phenotyping.

At birth (P0), living and dead G3 pups were counted and any gross abnormalities noted. Pups were tail clipped and weighed at P14. G3 animals were weaned at 24 d, weighed and their length measured before they were subjected to the wire hang and negative geotaxis assays [38]. Animals were then placed in a SHIRPA arena and activity measured for 30 s by counting the number of 11 × 11 cm squares visited, as defined by having all four paws in the same square at the same time. The gait and general behavior of the animals were also monitored. Animals were given a visual inspection to detect craniofacial or limb abnormalities.

### RT-PCR and standard PCR genotyping.

RNA was extracted and RT-PCR performed as described [8]. Transposase transgene genotyping was done using standard techniques on tail biopsy DNA. Individual insertion genotyping was done as previously described using three-primer PCR [39]. Primer sequences for all PCR reactions are available upon request.

### Immunohistochemistry.

Sections were cut from frozen muscle on a Tissue Tek II microtome/cryostat (Miles Scientific, Naperville, Illinois), fixed, and stored at −20 °C until use. Slides were air dried followed by incubation in permeabilizing/blocking solution (P/BS) (0.12% BSA, 0.12% nonfat dry milk, 0.1% Triton X-100 in phosphate-buffered saline [PBS]) plus 5% normal goat serum for 1 h at 4 °C and then rinsed three times in PBS. SC-71 (used as an undiluted hybridoma supernatant) reacts with MyHC-IIa *(Myh2),* NCL-MHCs (Novocastra Laboratories Newcastle-upon-Tyne, United Kingdom, 1:50 dilution) reacts with MyHC-I *(Myh7),* and Anti-Laminin (Sigma-Aldrich, St. Louis, Missouri, United States, 1:200 dilution) aided in muscle fiber identification. Primary antibody incubations were carried out at room temperature for 2 h, washed twice with PBS and incubated twice for 5 min in P/BS. Secondary antibody incubations were performed in P/BS at room temperature for 2 h using Goat-anti-mouse FITC- or TR-conjugated IgG (Jackson ImmunoResearch, West Grove, Pennsylvania, United States, 1:100 dilution) for Anti-MyHC detection and FITC-conjugated goat-anti-rabbit (Jackson ImmunoResearch, 1:100 dilution) for Anti-Laminin detection. Sections were washed 3 times with PBS, incubated twice for 5 min in PBS, and mounted in glycerol-gelatin (Sigma-Aldrich).

### Fluorescence in situ hybridization.

129Sv library BAC clones obtained complementary of The Wellcome Trust Sanger Institute (Cambridge, United Kingdom) and the pT2/GT3/tTA transposon-harboring plasmid [8], were labeled with biotin or digoxigenin, respectively, by nick translation as we have previously described [1]. Probes were detected using rhodamine-conjugated strepavidin or fluorescein-conjugated anti-digoxigenin antibody, respectively (Rainbow Scientific, Windsor, Connecticut, United States), as also described by Carlson et al [1].

### Representational oligonucleotide micro array.

High molecular weight genomic DNA was isolated from mouse brain using the Puregene Cell & Tissue kit from Gentra Systems (Minneapolis, Minnesota, United States, #D-5500A). For pedigrees W and AG, wild-type FVB/N strain DNA was used as a reference sample. BglII genomic representations, and Cy3-dCTP (reference DNA) or Cy5-dCTP (DNA of interest) incorporation, hybridization, and washing conditions were done as described recently [18]. Hybridizations were carried out on arrays bearing 85,000 oligonucleotides (NimbleGen, Systems, Madison, Wisconsin, United States). Design of the mouse array probes is described in Lakshmi et al. [40]. Slides were scanned with an Axon GenePix 4000B scanner (Axon Instruments, Union City, California, United States). Array data was imported into S-plus and was normalized as in Lucito et al. [18]. The moving averages of raw Cy5/Cy3 ratios are displayed in the figures. The genome position was determined from the UCSC GoldenPath browser (http://genome.ucsc.edu/cgi-bin/hgGateway?clade=vertebrate&org=Mouse&db=mm5) (May 2004 Assembly).

## Supporting Information

Table S1 Positions of T2/GT3/tTA Insertions into Mouse Chromosomes Linker-mediated PCR was used to determine the positions of 175 gene-trap tTA insertions after mobilization from the GT3A Chromosome-11 donor site. Sorted by chromosome and position, the insertions in the balanced region between *Trp53* and *Wnt3* are highlighted in blue.
(101 KB PDF)

Table S2 Summarized Somatic Fluorescence In Situ Hybridization Data FISH was performed on splenic lymphocytes of a GT3A; RosaSB11 mouse. Metaphase and interphase cells were screened for chromosome abnormalities using the same probes as shown in Figure 6. The table summarizes the percentages of normal and abnormal cells detected for each probe.
(21 KB XLS)

### Accession Numbers

Mouse Genome Informatics (http://www.informatics.jax.org) accession numbers for genes mentioned in the text include Car10 (MGI:2144598), Myh2 (MGI:1339710), syntaxin binding protein 4 gene (MGI:1342296), mouse homologs of a yeast cytochrome C oxidase assembly protein (MGI:1917052), Tom1l1 (MGI:1919193), monocyte to macrophage differentiation-associated gene (MGI:1914718), and hepatic leukemia factor (MGI:96108). The RefSeq (http://www.ncbi.nlm.nih.gov/RefSeq) peptide accession number for MyHC-IIa is NP_001034634. Additional accession number information for potential gene disruptions may be found in Table S1.

## Abbreviations

BAC: bacterial artificial chromosome
*Car10*: mouse carbonic anhydrase 10 gene
ENU: *N*-ethyl-*N*-nitrosourea
FISH: fluorescence in situ hybridization
*Myh2*: myosin heavy chain 2 gene
MyHC-IIa: myosin heavy chain IIa peptide
ROMA: representational oligonucleotide micro array
SB: *Sleeping Beauty*
*Tom1/1*: chicken target of myb1-like 1

## References

[pgen-0020156-b001] Carlson CM, Dupuy AJ, Fritz S, Roberg-Perez KJ, Fletcher CF (2003). Transposon mutagenesis of the mouse germline. Genetics.

[pgen-0020156-b002] Dupuy AJ, Fritz S, Largaespada DA (2001). Transposition and gene disruption in the male germline of the mouse. Genesis.

[pgen-0020156-b003] Horie K, Kuroiwa A, Ikawa M, Okabe M, Kondoh G (2001). Efficient chromosomal transposition of a Tc1/mariner- like transposon Sleeping Beauty in mice. Proc Natl Acad Sci U S A.

[pgen-0020156-b004] Horie K, Yusa K, Yae K, Odajima J, Fischer SE (2003). Characterization of Sleeping Beauty transposition and its application to genetic screening in mice. Mol Cell Biol.

[pgen-0020156-b005] Keng VW, Yae K, Hayakawa T, Mizuno S, Uno Y (2005). Region-specific saturation germline mutagenesis in mice using the Sleeping Beauty transposon system. Nat Methods.

[pgen-0020156-b006] Collier LS, Carlson CM, Ravimohan S, Dupuy AJ, Largaespada DA (2005). Cancer gene discovery in solid tumours using transposon-based somatic mutagenesis in the mouse. Nature.

[pgen-0020156-b007] Dupuy AJ, Akagi K, Largaespada DA, Copeland NG, Jenkins NA (2005). Mammalian mutagenesis using a highly mobile somatic Sleeping Beauty transposon system. Nature.

[pgen-0020156-b008] Geurts AM, Wilber A, Carlson CM, Lobitz PD, Clark KJ (2006). Conditional gene expression in the mouse using a Sleeping Beauty gene-trap transposon. BMC Biotechnol.

[pgen-0020156-b009] Hentges KE, Justice MJ (2004). Checks and balancers: Balancer chromosomes to facilitate genome annotation. Trends Genet.

[pgen-0020156-b010] Zheng B, Sage M, Cai WW, Thompson DM, Tavsanli BC (1999). Engineering a mouse balancer chromosome. Nat Genet.

[pgen-0020156-b011] Kile BT, Hentges KE, Clark AT, Nakamura H, Salinger AP (2003). Functional genetic analysis of mouse chromosome 11. Nature.

[pgen-0020156-b012] Hentges KE, Nakamura H, Furuta Y, Yu Y, Thompson DM (2006). Novel lethal mouse mutants produced in balancer chromosome screens. Gene Expr Patterns.

[pgen-0020156-b013] Fischer SE, Wienholds E, Plasterk RH (2001). Regulated transposition of a fish transposon in the mouse germ line. Proc Natl Acad Sci U S A.

[pgen-0020156-b014] Allen DL, Harrison BC, Leinwand LA (2000). Inactivation of myosin heavy chain genes in the mouse: Diverse and unexpected phenotypes. Microsc Res Tech.

[pgen-0020156-b015] Acakpo-Satchivi LJ, Edelmann W, Sartorius C, Lu BD, Wahr PA (1997). Growth and muscle defects in mice lacking adult myosin heavy chain genes. J Cell Biol.

[pgen-0020156-b016] Sartorius CA, Lu BD, Acakpo-Satchivi L, Jacobsen RP, Byrnes WC (1998). Myosin heavy chains IIa and IId are functionally distinct in the mouse. J Cell Biol.

[pgen-0020156-b017] Geurts AM, Yang Y, Clark KJ, Liu G, Cui Z (2003). Gene transfer into genomes of human cells by the sleeping beauty transposon system. Mol Ther.

[pgen-0020156-b018] Lucito R, Healy J, Alexander J, Reiner A, Esposito D (2003). Representational oligonucleotide microarray analysis: A high-resolution method to detect genome copy number variation. Genome Res.

[pgen-0020156-b019] Zhang J, Peterson T (1999). Genome rearrangements by nonlinear transposons in maize. Genetics.

[pgen-0020156-b020] Zhang J, Peterson T (2004). Transposition of reversed Ac element ends generates chromosome rearrangements in maize. Genetics.

[pgen-0020156-b021] Zhang J, Peterson T (2005). A segmental deletion series generated by sister-chromatid transposition of Ac transposable elements in maize. Genetics.

[pgen-0020156-b022] Martin C, Lister C (1989). Genome juggling by transposons: Tam3-induced rearrangements in Antirrhinum majus. Dev Genet.

[pgen-0020156-b023] Moerman DG, Kiff JE, Waterston RH (1991). Germline excision of the transposable element Tc1 in C. elegans. Nucleic Acids Res.

[pgen-0020156-b024] Engels WR, Preston CR (1984). Formation of chromosome rearrangements by P factors in Drosophila. Genetics.

[pgen-0020156-b025] Gray YH (2000). It takes two transposons to tango: Transposable-element-mediated chromosomal rearrangements. Trends Genet.

[pgen-0020156-b026] Hallet B, Sherratt DJ (1997). Transposition and site-specific recombination: Adapting DNA cut-and-paste mechanisms to a variety of genetic rearrangements. FEMS Microbiol Rev.

[pgen-0020156-b027] Zhang J, Zhang F, Peterson T (2006). Transposition of reversed Ac element ends generates novel chimeric genes in maize. PLoS Genet.

[pgen-0020156-b028] Drabek D, Zagoraiou L, deWit T, Langeveld A, Roumpaki C (2003). Transposition of the Drosophila hydei Minos transposon in the mouse germ line. Genomics.

[pgen-0020156-b029] Ding S, Wu X, Li G, Han M, Zhuang Y (2005). Efficient transposition of the piggyBac (PB) transposon in mammalian cells and mice. Cell.

[pgen-0020156-b030] Nadeau JH, Taylor BA (1984). Lengths of chromosomal segments conserved since divergence of man and mouse. Proc Natl Acad Sci U S A.

[pgen-0020156-b031] Kidwell MG, Lisch D (1997). Transposable elements as sources of variation in animals and plants. Proc Natl Acad Sci U S A.

[pgen-0020156-b032] Medstrand P, van de Lagemaat LN, Dunn CA, Landry JR, Svenback D (2005). Impact of transposable elements on the evolution of mammalian gene regulation. Cytogenet Genome Res.

[pgen-0020156-b033] Smit AF (1999). Interspersed repeats and other mementos of transposable elements in mammalian genomes. Curr Opin Genet Dev.

[pgen-0020156-b034] Lander ES, Linton LM, Birren B, Nusbaum C, Zody MC (2001). Initial sequencing and analysis of the human genome. Nature.

[pgen-0020156-b035] Waterston RH, Lindblad-Toh K, Birney E, Rogers J, Abril JF (2002). Initial sequencing and comparative analysis of the mouse genome. Nature.

[pgen-0020156-b036] Lyttle TW, Haymer DS (1992). The role of the transposable element hobo in the origin of endemic inversions in wild populations of Drosophila melanogaster. Genetica.

[pgen-0020156-b037] Ivics Z, Hackett PB, Plasterk RH, Izsvak Z (1997). Molecular reconstruction of Sleeping Beauty, a Tc1-like transposon from fish, and its transposition in human cells. Cell.

[pgen-0020156-b038] Rogers DC, Fisher EM, Brown SD, Peters J, Hunter AJ (1997). Behavioral and functional analysis of mouse phenotype: SHIRPA, a proposed protocol for comprehensive phenotype assessment. Mamm Genome.

[pgen-0020156-b039] Dupuy AJ, Clark K, Carlson CM, Fritz S, Davidson AE (2002). Mammalian germ-line transgenesis by transposition. Proc Natl Acad Sci U S A.

[pgen-0020156-b040] Lakshmi B, Hall IM, Egan C, Alexander J, Leotta A (2006). Mouse genomic representational oligonucleotide microarray analysis: Detection of copy number variations in normal and tumor specimens. Proc Natl Acad Sci U S A.

