# Supplementary material for: Gene Mutations and Genomic Rearrangements in the Mouse as a Result of Transposon Mobilization from Chromosomal Concatemers
Source: PLoS Genet. 2006 Sep 29;2(9):e156. doi: 10.1371/journal.pgen.0020156 (PMC1584263; doi:10.1371/journal.pgen.0020156)
Supplement: Table S1 — Linker-mediated PCR was used to determine the positions of 175 gene-trap tTA insertions after mobilization from the GT3A Chromosome-11 donor site. Sorted by chromosome and position, the insertions in the balanced region between Trp53 and Wnt3 are highlighted in blue. (101 KB PDF) [file pgen.0020156.st001.pdf]

Table S1: Insertion data

seq ID	identity	Chr	position[a]	hit[b]	Orient.[c]	description
03A-0118	100	1	16,702,060	ENSMUSESTG	v5	
03A-0142	100	1	45,259,459	nothing		
03A-0273	100	1	66,279,744	GENSCAN		
03A-0227	95+	1	125,574,520	Gpr39	tTA	G protein-coupled receptor 39 [Source:MarkerSymbol;Acc:MGI:1918361]
03A-0075	100	1	180,102,143	Csc42bpa	tTA	Cdc42 binding protein kinase alpha [Source:MarkerSymbol;Acc:MGI:2441841]
03A-0233	91+	2	22,353,080	Myo3a	tTA	Myosin IIIA (EC 2.7.1.37). [Source:Uniprot/SWISSPROT;Acc:Q8K3H5]
03A-0228	100	2	40,407,579	nothing		
03A-0112	100	2	55,436,182	kcnj3	tTA	G protein-activated inward rectifier potassium channel 1 (GIRK1) (Potassium channel, inwardly rectifying, subfamily J, member 3) (Inward rectifier K+ channel Kir3.1) (KGA)
03A-0224	100	2	87,753,810	nothing		
03A-0058	98+	2	131,063,998	Adra1d	tTA	adrenergic receptor, alpha 1d [Source:MarkerSymbol;Acc:MGI:106673]
03A-0125	99+	2	148,046,460	nothing		
03A-0272	100	3	22,850,979	nothing		
03A-0241	100	4	122,663,707	Macf1	tTA	Microtubule-actin crosslinking factor 1 (Actin cross-linking family 7). [Source:Uniprot/SWISSPROT;Acc:Q9QXZ0]
03A-0162	100	5	129,871,376	Caln1	tTA	Calneuron 1. [Source:Uniprot/SWISSPROT;Acc:Q9JJG7]
03A-0130	100	5	143,554,337	nothing		
03A-0231	100	5	146,879,116	ENSMUSESTG	v5	
03A-0073	100	5	149,611,803	GENSCAN		
03A-0320	91+	6	6,130,080	GENSCAN		
03A-0329	100	6	61,300,534	GENSCAN		
03A-0124	100	6	62,658,593	nothing		
03A-0288	100	6	62,658,594	nothing		
03A-0183	100	6	147,279,984	Itpr5	v5	Inositol 1,4,5-trisphosphate receptor type 2 (Type 2 inositol 1,4,5- trisphosphate receptor) (Type 2 InsP3 receptor) (IP3 receptor isoform 2) (InsP3R2) (Inositol 1,4,5-trisphosphate type V receptor) (Fragments). [Source:Uniprot/SWISSPROT;Acc:Q9Z329]
03A-0146	91+	7	41,742,312	GENSCAN		
03A-0167	100	7	90,754,185	Odz4	v5	odd Oz/ten-m homolog 4 (Drosophila) [Source:MarkerSymbol;Acc:MGI:2447063]
03A-0117	100	7	96,783,421	nothing		
03A-0091	100	8	48,422,540	nothing		
03A-0229	100	8	90,228,185	Chd9	v5	chromodomain helicase DNA binding protein 9 [Source:MarkerSymbol;Acc:MGI:1924001]
03A-0077	100	9	9,226,929	Q9D333	v5	Adult male colon cDNA, RIKEN full-length enriched library, clone:9030420J04 product:RIKEN cDNA 9030420J04. Source: Uniprot/SPTREMBL Q9D333
03A-0128	100	10	22,570,444	ENSMUSG	v5	
03A-0154	100	10	22,570,444	ENSMUSG	v5	
03A-0153	98+	10	50,206,677	LINE element		
03A-0232	100	10	100,245,703	GENSCAN		
03A-0132	100	10	102,520,111	nothing		
03A-0105	100	10	110,229,348	nothing		
03A-0359	100	10	112,582,818	GENSCAN		
03A-0029	97+	10	113,468,399	nothing		
03A-0286	99+	11	20,386,036	GENSCAN		
03A-0225	98+	11	32,726,257	GENSCAN		
03A-0296	100	11	38,387,305	nothing		

Table S1 ***Continued***

seq ID	identity	Chr	position[a]	hit[b]	Orient.[c]	description
03A-0126	100	11	38,943,644	GENSCAN		
03A-0193	100	11	39,119,812	ENSMUSG	tTA	
03A-0325	100	11	39,633,085	GENSCAN		
03A-0057	99+	11	39,963,249	RP23-31O3.1		No description
03A-0279	100	11	44,820,144	nothing		
03A-0180	100	11	48,150,349	GENSCAN		
03A-0234	100	11	48,219,501	GENSCAN		
03A-0310	100	11	54,289,495	Rapgef6	tTA	Rap guanine nucleotide exchange factor (GEF) 6 [Source:MarkerSymbol;Acc:MGI:2384761]
03A-0355	100	11	62,027,478	Ttc19	tTA	tetratricopeptide repeat domain 19 [Source:MarkerSymbol;Acc:MGI:1920045]
03A-0220	100	11	62,042,119	Ttc19 (3'UTR) and Ncor1 (3'UTR)	v5 (Ttc19) and tTA (Ncor1)	tetratricopeptide repeat domain 19 [Source:MarkerSymbol;Acc:MGI:1920045] nuclear receptor co-repressor 1
03A-0168	100	11	63,090,815	GENSCAN		
03A-0376	100	11	64,482,102	GENSCAN		
03A-0375	99+	11	64,570,290	GENSCAN		
03A-0063	100	11	66,910,670	Myh2	v5	myosin, heavy polypeptide 2, skeletal muscle, adult
03A-0314	94+	11	73,993,080	NM_011002		
03A-0321	100	11	79,485,485	RP23-307I20.1	v5	
03A-0098	100	11	80,135,251	GENSCAN		
03A-0349	100	11	81,418,900	Accn1 AND GENSCAN	v5 (Accn1)	amiloride-sensitive cation channel 1, neuronal (degenerin) [Source:MarkerSymbol;Acc:MGI:1100867]
03A-0366	99+	11	81,429,851	Accn1 AND GENSCAN	tTA (Accn1)	amiloride-sensitive cation channel 1, neuronal (degenerin) [Source:MarkerSymbol;Acc:MGI:1100867]
03A-0284	100	11	84,675,962	nothing		
03A-0370	100	11	88,050,914	nothing		
03A-0163	100	11	88,250,423	Msih2, GENSCAN	v5 (Mshi2)	Musashi homolog 2 (Drosophila) [Source:MarkerSymbol;Acc:MGI:1923876]
03A-0212	100	11	88,283,913	Msih2	tTA	Musashi homolog 2 (Drosophila) [Source:MarkerSymbol;Acc:MGI:1923876]
03A-0332	100	11	88,565,723	GENSCAN		
03A-0274	100	11	88,814,203	GENSCAN and RP23-176J13.6 (3'UTR)	tTA (RP23-176J13.6)	
03A-0204	99+	11	89,247,242	Q5SRA9_MOUSE	tTA	Novel protein (Likely ortholog to homo sapiens FLJ38335) (Fragment). Source: Uniprot/SPTREMBL Q5SRA9
03A-0095	100	11	89,315,247	Q5SRA9_MOUSE	v5	Novel protein (Likely ortholog to homo sapiens FLJ38335) (Fragment). Source: Uniprot/SPTREMBL Q5SRA9
03A-0265	100	11	89,322,258	Q5SRA9_MOUSE	v6	Novel protein (Likely ortholog to homo sapiens FLJ38335) (Fragment). Source: Uniprot/SPTREMBL Q5SRA9
03A-0381	96+	11	89,337,401	Q5SRA9_MOUSE	tTA	Novel protein (Likely ortholog to homo sapiens FLJ38335) (Fragment). Source: Uniprot/SPTREMBL Q5SRA9
03A-0353	100	11	89,520,744	GENSCAN		
03A-0114	100	11	89,733,741	GENSCAN		
03A-0129	97+	11	89,737,545	GENSCAN		
03A-0304	100	11	89,867,873	GENSCAN		
03A-0275	98+	11	89,872,893	GENSCAN		
03A-0093	97+	11	89,946,442	nothing		
03A-0159	100	11	89,982,392	GENSCAN		

Table S1 *Continued*						
**seq ID**	**identity**	**Chr**	**position[a]**	**hit[b]**	**Orient.[c]**	**description**
03A-0280	100	11	90,065,494	Mmd	tTA	monocyte to macrophage differentiation-associated
03A-0292	99+	11	90,118,225	nothing		
03A-0315	100	11	90,138,937	nothing		
03A-0216	100	11	90,161,624	Hlf (5'UTR)	v5	hepatic leukemia factor [Source:MarkerSymbol;Acc:MGI:96108]
03A-0059	100	11	90,191,017	Hlf	tTA	hepatic leukemia factor [Source:MarkerSymbol;Acc:MGI:96108]
03A-0061	100	11	90,197,484	Hlf	tTA	hepatic leukemia factor [Source:MarkerSymbol;Acc:MGI:96108]
03A-0076	100	11	90,336,388	Stxbp4	tTA	syntaxin binding protein 4 [Source:MarkerSymbol;Acc:MGI:1342296]
03A-0367	100	11	90,346,182	Stxbp4	v5	syntaxin binding protein 4 [Source:MarkerSymbol;Acc:MGI:1342296]
03A-0377	99+	11	90,409,879	Stxbp4	v5	syntaxin binding protein 4 [Source:MarkerSymbol;Acc:MGI:1342296]
03A-0297	99+	11	90,468,899	Srcasm and Tom1l1	v5	Src activating and signaling molecule and Target of myb1-like 1 (chicken) [Source:MarkerSymbol;Acc:MGI:1919193]
03A-0186	100	11	90,540,640	nothing		
03A-0122	100	11	90,547,619	nothing		
03A-0036	100	11	90,576,639	GENSCAN		
03A-0192	100	11	90,641,443	RP23-219D8.1	tTA	
03A-0291	100	11	90,664,024	RP23-219D8.1	v5	
03A-0155	100	11	90,795,830	GENSCAN		
03A-0215	100	11	90,796,368	GENSCAN		
03A-0127	100	11	90,855,319	GENSCAN		
03A-0334	100	11	91,233,208	GENSCAN		
03A-0011	100	11	91,326,753	nothing		
03A-0119	99+	11	91,439,069	nothing		
03A-0335	94+	11	91,512,812	GENSCAN		
03A-0078	98+	11	91,624,576	nothing		
03A-0207	100	11	91,788,257	GENSCAN		
03A-0181	100	11	91,828,307	GENSCAN		
03A-0384	98+	11	91,867,809	GENSCAN		
03A-0308	100	11	91,932,735	nothing		
03A-0100	100	11	92,136,825	nothing		
03A-0351	100	11	92,320,484	nothing		
03A-0071	100	11	92,472,167	GENSCAN		
03A-0089	100	11	92,720,039	GENSCAN		
03A-0222	100	11	92,723,469	GENSCAN		
03A-0282	100	11	92,757,285	nothing		
03A-0033	100	11	92,982,091	Car10	tTA	carbonic anhydrase 10 [Source:MarkerSymbol;Acc:MGI:1919855]
03A-0213	100	11	93,190,010	Car10	tTA	carbonic anhydrase 10 [Source:MarkerSymbol;Acc:MGI:1919855]
03A-0210	100	11	93,272,269	Car10	v5	carbonic anhydrase 10 [Source:MarkerSymbol;Acc:MGI:1919855]
03A-0382	99+	11	93,393,216	Car10	tTA	carbonic anhydrase 10 [Source:MarkerSymbol;Acc:MGI:1919855]
03A-0383	100	11	93,494,634	nothing		
03A-0103	98+	11	93,525,508	nothing		
03A-0356	100	11	93,635,449	nothing		
03A-0104	97+	11	93,973,736	nothing	v5	
03A-0085	100	11	94,306,736	BC018371	v5	cDNA sequence BC018371 [Source:MarkerSymbol;Acc:MGI:2388287]
03A-0211	100	11	95,259,178	Spop	tTA	speckle-type POZ protein. [Source: RefSeq (NM_025287)]

Table S1 *Continued*						
**seq ID**	**identity**	**Chr**	**position[a]**	**hit[b]**	**Orient.[c]**	**description**
03A-0305	100	11	96,066,602	nothing		
03A-0052	100	11	96,075,928	nothing		
03A-0026	97+	11	99,081,093	GENSCAN		
03A-0123	99+	11	100,107,468	nothing	v5	
03A-0018	100	11	101,198,955	G6pc (3'UTR)		glucose-6-phosphatase, catalytic [Source:MarkerSymbol;Acc:MGI
03A-0217	100	11	104,481,070	IT_B3 MOUSE	tTA	Integrin beta-3 precursor (Platelet membrane glycoprotein IIIa) (GPIIIa) (CD61 antigen). [Source:Uniprot/SWISSPROT;Acc:O54890]
03A-0087	100	11	104,662,430	nothing		
03A-0309	100	11	105,207,608	Rnf190	tTA	ring finger protein 190 [Source:MarkerSymbol;Acc:MGI:2443469]
03A-0298	100	11	105,782,146	nothing		
03A-0102	100	11	109,524,360	nothing		
03A-0348	100	11	109,812,822	AbcaB8	tTA	ATP-binding cassette, sub-family A (ABC1), member 8b [Source:MarkerSymbol;Acc:MGI:1351668]
03A-0157	96+	11	110,949,387	nothing		
03A-0330	95+	11	110,949,394	nothing		
03A-0111	100	11	111,431,807	nothing		
03A-0306	100	11	112,040,772	GENSCAN	v5	
03A-0363	100	12	7,467,396	1110057K04Rik RIKEN	tTA	cDNA 1110057K04 gene (1110057K04Rik), mRNA Source: RefSeq_dna NM_172401
03A-0198	100	12	88,198,854	nothing		
03A-0045	100	13	14,200,635	GENSCAN		
03A-0326	99+	13	14,200,635	GENSCAN		
03A-0189	100	13	57,440,963	nothing		
03A-0221	99+	13	57,593,094	Ntrk2	v5	neurotrophic tyrosine kinase, receptor, type 2 [Source:MarkerSymbol;Acc:MGI:97384]
03A-0107	99+	13	64,838,138	Adcy2	v5	adenylate cyclase 2 [Source:MarkerSymbol;Acc:MGI:99676]
03A-0175	99+	13	81,279,049	nothing		
03A-0062	99+	13	84,771,595	nothing		(close to edil3)
03A-0281	100	14	18,955,686	C030002J06Rik	v5	cDNA C030002J06 gene (C030002J06Rik), mRNA Source: RefSeq_dna NM_176988
03A-0137	99+	14	18,955,879	C030002J06Rik	v5	RIKEN cDNA C030002J06 gene (C030002J06Rik), mRNA Source: RefSeq_dna NM_176988
03A-0246	100	14	45,890,049	ENSMUSESTG	v5	
03A-0148	98+	15	16,416,966	LINE element		
03A-0244	96+	15	75,118,722	GENSCAN		
03A-0311	99+	15	78,355,502	Csf2rb2	tTA	colony stimulating factor 2 receptor, beta 2, low-affinity (granulocyte-macrophage) [Source:MarkerSymbol;Acc:MGI:1339760]
03A-0094	100	15	96,087,105	nothing		
03A-0268	99+	16	14,495,274	Prkdc	v5	protein kinase, DNA activated, catalytic polypeptide [Source:MarkerSymbol;Acc:MGI:104779]
03A-0188	100	16	18,177,639	nothing		
03A-0247	100	16	59,361,301	Epha6, GENSCAN	v5	Ephrin type-A receptor 6 precursor (EC 2.7.1.112) (Tyrosine-protein kinase receptor EHK-2) (EPH homology kinase-2). [Source:Uniprot/SWISSPROT;Acc:Q62413]
03A-0301	100	17	59,209,581	nothing		
03A-0316	98+	17	80,342,477	ENSMUSG00000066926	tTA	
03A-0040	99+	17	80,342,477	ENSMUSG00000066926	v5	

Table S1 *Continued*

seq ID	identity	Chr	position[a]	hit[b]	Orient.[c]	description
03A-0043	100	17	89,359,599	nothing		
03A-0323	100	17	89,783,047	GENSCAN		
03A-0039	100	17	89,783,049	GENSCAN		
03A-0327	100	17	89,946,909	nothing		
03A-0333	100	17	90,099,545	nothing		
03A-0067	100	17	91,800,652	nothing		
03A-0243	100	18	12,698,929	Lama3	v5	Laminin alpha-3 chain precursor (Nicein alpha subunit). [Source:Uniprot/SWISSPROT;Acc:Q61789]
03A-0096	100	18	35,406,762	Catna1	tTA	Alpha-1 catenin (102 kDa cadherin-associated protein) (CAP102) (Alpha E-catenin). [Source:Uniprot/SWISSPROT;Acc:P26231]
03A-0064	100	18	61,764,006	ENSMUSESTG	tTA	
03A-0055	100	18	72,913,663	GENSCAN		
03A-0184	99+	19	10,699,206	Ms4a4c	tTA	membrane-spanning 4-domains, subfamily A, member 4C [Source:MarkerSymbol;Acc:MGI:1927656]

[a] Reported is the coordinate of the T of the target TA dinucleotide for SB transposon insertion, according to the ENSEMBL m34 NCBI build, insertions within the balanced region between *Wnt3* and *Trp52* are highlighted in blue

[b] Reported is the insertion into known, predicted (ENSMUSG, GENSCAN), or expressed sequence tag or cDNA (ENSMUSESTG)

[c] Reported is the orientation which the gene-trap tTA (Fig. 1A) inserted with respect to transcriptional orientation of the endogenous gene.
